# Clinical Remarks on Sympathetic Inflammation of the Eyeball, Its Danger and the Means of Arresting It

**Published:** 1862-09

**Authors:** Haynes Walton

**Affiliations:** Surgeon to the Hospital


### CLINICAL REMARKS ON SYMPATHETIC INFLAMMATION OF THE EYEBALL, ITS DANGER, AND THE MEANS OF ARRESTING IT.

*Made at the Central London Ophthalmic Hospital.*

BY HAYNES WALTON, Surgeon to the Hospital.

The subject is among those in this department of medicine that modern observation has thoroughly recognized, and investigation and rational experiment have found a remedy for. Sympathetic inflammation, or sympathetic ophthalmitis, may arise out of any circumstances that produce disorganization of the eyeball. It is most commonly, however, seen when an eye has been spoiled by wounds. This is the usual course of things. An eye is wounded, perhaps severely, and the lens has escaped, or a portion of the vitreous humour; or, perhaps, the cornea only has been penetrated, and the iris, or the lens wounded. The acute and primary inflammatory attack is subdued, and chronic disease supervenes. The heretofore sound eye gets intolerant to light, the first common result; impaired vision in some form is the next bad omen. Loss of focal adjustment, incapability of sustaining vision on minute objects, loss of definition (generally called feeble sight), muscæ, spectra, flashes, stars, inflammatory action, loss of pupillary movements, change of iris color, softening of the eyeball, and shrinking, are the later manifestations. Thus it would seem that the morbid action travels from the retina forwards. Ultimately, all the ocular tissues are involved, and atrophy ensues. There may be varieties in the subjective and objective symptoms; and there may be no pain, or it may exist with great severity.

The sympathetic action is imminent, so long as any irritation produced by the traumatic disease lingers, so that it may be developed in a few weeks, or not for years. It cannot be said that any peculiar form of wound, or the injury of any particular tissue, excites that kind of action which develops the sympathy; as blows without breach of surface, or chemical injuries, may cause it.

Inflammatory affections producing disorganization of the eye may induce sympathetic disease. I have seen the greater number of cases arising from staphyloma of the cornea and the sclerotica—that is, general enlargement of the eyeball—the result of purulent ophthalmia in infancy, than any other cause.

The diagnosis is by no means obscure, and in traumatic cases it is most easy. The eye primarily injured or diseased, always manifests symptoms of irritation or disturbance; and their is evidence of acute or chronic inflammation. These may be slight; but they are to be discerned with care. There is always soreness under touch. So far as I know, vision is invariably extinct. If, then, a patient who has lost an eye from accident or disease, were to apply to me on account of the eye heretofore well, but now attacked with any of the symptoms that I have pointed out of sympathetic derangement, I should examine the eye primarily injured; and if I discovered any morbid action in progress, any of those states which are connected with, or arise out of, what is called inflammation, I should say that I had before me a case of sympathetic ophthalmitis.

There are two errors into which you may fall, but they are easily avoided when you are on your guard. Do not, then, mistake for sympathy what is merely the same disease that has appeared in the one eye, and is secondary only in the order of time. Remember that the destruction of one eye, from any cause, may be followed, although the occurrence is not common, by the loss of the power of the retina in the other, and this without the least trace of any active symptom in that which was hurt. Precisely the same thing may occur after the one eyeball has collapsed, or has even been extirpated, so that sympathetic inflammation can have nothing to do with it.

The treatment is definite and sure; but is not to be found in general remedies, local applications, nor any dietary system. Nothing of the kind can be depended on; the affection can be stopped, or subdued, only by surgical treatment. A portion of the originally diseased eyeball must be removed, whereby the products that have set up the irritation, or the cretaceous, or ossified tissue, which has acted as a foreign body, may be got rid of; or extirpation resorted to. The practice works wonders when done early. If adopted before the sympathetic action has induced palpable structural charges, it will be all effectual. At later stages it may arrest progress, and stay the destruction. Even when the pupil has become adherent to the capsule of the lens, and the iris dull, I have known a check.

As a rule, the removal of a portion of the eyeball, "absciossion," is adapted to those cases in which the eye has been wounded in the front part, and the abnormal changes limited to that portion of the organ. When the whole eyeball evidently is diseased, and especially when there is distension of the sclerotic coat, or general enlargement, "extirpation" is the more adapted.

I perform abscission in this manner. The eyelids having been retracted, I transfix the cornea, or whatever remains of it, or the staphyloma, if there be one, with an ordinary tenaculum, and cut it off with a long and narrow scalpel, gently and quickly. It may be necessary to make the amputation a little behind the cornea, and then the iris, or whatever remains of it, is taken away. When the lens is present, whether opaque or not, it ought to be removed. An attempt should be made by gently manipulating and rapid closing of the eyelids, to save as much as possible of the vitreous humour. I now place a ball of cotton wool or a pledget of lint quickly over the eyelids, maintain it with a bandage, and keep the eye so bound for two or three days. Afterwards, I apply strips of plaster. There is no more important part in the proceeding than this, without which being properly done there may be troublesome bleeding and long convalescence. Healing is effected by the cicatrizing of the surface, and its rapidity depends on the healthiness of the vitreous humour. Among the advantages of "abscission," may be mentioned that it admits of the most perfect adaptation of an artificial eye. This is through the stump that is left.

"Extirpation" should be done within the "ocular sheath" of the eyeball. For much that is interesting with regard to this sheath or tissue, of which the existence was only made out a few years ago by Dr. O'Ferrall, of Dublin, I beg to refer you to my work on the *Surgical Diseases of the Eye*, second edition. This operation would be called a more brilliant one than "abscission;" and there can be no doubt that, although the proceeding, so far as the practial surgery is concerned, is more prolonged and severe, the recovery may be more rapid, and the general effect on the system perhaps less. Yet I am quite sure that if the patient's ultimate welfare be considered, its adoption should be the rare exception.

I consider this to be the best manner of doing the extirpation. The eyelids having been separated by the silver wire retractor, the conjunctiva is dissected off with forceps and scissors close to its ocular attachment; the recti and oblique muscles then taken up severally with the hook, as in the operation for strabismus, and divided at their insertions; the sheath detached by a probe or hook, from the eyeball, which should now be drawn aside, and the optic nerve cut through. There is generally no bleeding; but should it occur, a compress and bandage must be employed. —*British Med. Journal.*

